# Quality of Life of Medical Students in China: A Study Using the WHOQOL-BREF

**DOI:** 10.1371/journal.pone.0049714

**Published:** 2012-11-27

**Authors:** Yang Zhang, Bo Qu, Shisi Lun, Dongbo Wang, Ying Guo, Jie Liu

**Affiliations:** 1 Research Centre for Medical Education, China Medical University, Shenyang, China; 2 Fourth Affiliated Hospital, China Medical University, Shenyang, China; 3 Department of Health Statistics, School of Public Health, China Medical University, Shenyang, China; Tehran University of Medical Sciences, Iran (Republic of Islamic)

## Abstract

**Objective:**

The aim of this study was to assess the quality of life (QOL) of medical students during their medical education and explore the influencing factors of the QOL of students.

**Methods:**

A cross-sectional study was conducted in June 2011. The study population was composed of 1686 medical students in years 1 to 5 at China Medical University. The Chinese version of WHOQOL-BREF instrument was used to assess the QOL of medical students. The reliability and validity of the questionnaire were assessed by Cronbach’s α coefficient and factor analysis respectively. The relationships between QOL and the factors including gender, academic year level, and specialty were examined using t-test or one-way ANOVA followed by Student-Newman–Keuls test. Statistic analysis was performed by SPSS 13.0.

**Results:**

The overall Cronbach’s α coefficient of the WHOQOL-BREF questionnaire was 0.731. The confirmatory factor analysis provided an acceptable fit to a four-factor model in the medical student sample. The scores of different academic years were significantly different in the psychological health and social relations domains (p<0.05). Third year students had the lowest scores in psychological health and social relations domains. The scores of different specialties had significant differences in psychological health and social relations domains (p<0.05). Students from clinical medicine had the highest scores. Gender, interest in the area of study, confidence in career development, hometown location, and physical exercise were significantly associated with the quality of life of students in some domains (p<0.05).

**Conclusions:**

The WHOQOL-BREF was reliable and valid in the assessment of the QOL of Chinese medical students. In order to cope with the influencing factors of the QOL, medical schools should carry out curriculum innovation and give the necessary support for medical students, especially for 3^rd^ year students.

## Introduction

With the rapid development of China’s economy, stemming from 1995, China gradually implemented a reform on higher education, changing the grasp of government limitations on education. This allowed academia and educational institutions to gain control over their own administration, form a new economic management, and improve their educational efficiency. However, the diminishment of government intervention and funding forced higher education institutions to increase their enrollment numbers to obtain the necessary financial support that once came from the government. With the increasing enrollment of universities in China since 1999, the volume of medical students has expanded rapidly [1]. This results in many problems, such as a serious shortage of logistical services and teaching facilities. According to statistics taken by the Chinese government, the enrollment number of medical students, including junior college students of medicine, had increased from 75188 in 1998 to 533618 in 2010 [2]. Chinese medical students face various problems, such as greater pressure on finding post-graduation employment [3], poor living environments and learning conditions, and other psychological issues [4], which have negative impacts on the quality of life (QOL) of medical students.

Quality of life is defined by the WHO as, “an individual’s perception of their position in life in the context of the culture and value systems in which they live, and in relation to their goals, expectations, standards and concerns” [5]. Great attention has been focused on different populations ever since the concept of quality of life has become widely accepted by society [6], [7]. Although previous studies have assessed the QOL of medical students with the WHOQOL-BREF [8], [9], few studies focus on Chinese medical students.

Studies have reported that medical education and training have a negative impact on students’ physical and mental health [10], [11]. Medical education is always long in duration and consists of great academic pressure and narrow professional employment opportunities. Some medical students with poor academic and professional performance are failed for the above issues. Compared to the general population, medical students are more susceptible to stress [12], burning out [10], [13], depression [14] and anxiety [15]. There may be several factors for this situation, such as academic courses and training [16], [17], contact with diseases and death [18]–[20].

The quality of life (QOL) of Chinese medical students is of growing concern to educators and administrators in recent years [21], [22]. Some studies on the QOL of medical students were reported with different instruments [9], [16], such as the WHOQOL-BREF and the SF-36. Previous studies using the Chinese version of WHOQOL-BREF focused mainly on different populations which provided good psychometric properties [23], [24]. Assessing the quality of life of medical students can inform us of their perspectives on health, current health conditions, and relevant factors. In the long run, promoting students’ well-being will benefit patients, the public, and the profession, in addition to the individual.

The aim of the study was to assess the QOL of Chinese medical students in years 1–5 of their medical training by using the Chinese version of the World Health Organization Quality of Life (WHOQOL-BREF) and to explore the influencing factors of the QOL of medical students.

## Methods

### Study Setting and Population

China Medical University follows a traditional Flexnerian curriculum. Medical training lasts 5 years and is divided into 2 years of basic sciences, 2 years of clinical medicine, and 1 year of internship. In the third and fourth years, students take courses including pathology, pharmacology, internal medicine, surgery, pediatrics and obstetrics. This is also when they have their first contact with patients in a hospital setting. Most medical students are admitted directly from high school.

A cross-sectional study was conducted in June 2011. Students were selected using cluster sampling method. We sent copies of the questionnaires to student union leaders who were fully trained in the questionnaire and surveying process and distributed the questionnaires to all students simultaneously on the same day. The students were given 10 minutes to complete the questionnaire independently. If there were any uncertainties regarding the questionnaire, students could consult the surveyors at any time.

According to government regulation, China Medical University is limited to a fixed enrollment number of students each year. The sample included 1768 medical students from 56 classes of years 1 to 5 at China Medical University: there were 8 classes from clinical medicine, 2 classes from preventive medicine, and 2 classes from nursing for year 1 to year 4. As some 5th year clinical students had their medical training in other cities, we chose 4 classes from clinical medicine, 2 classes from preventive medicine, and 2 classes from nursing for year 5. 1707 completed questionnaires were returned, and 1686 of these were valid. If more than four items in the questionnaire were missing, the questionnaire was excluded from the study.

### Instruments

Two instruments were used for data collection: (1) a socio-demographic questionnaire to obtain information on gender, grade, interest in the area of study, confidence in career development, and hometown location; and (2) the Chinese version of the WHOQOL-BREF questionnaire based on a brief version of the World Health Organization Quality of Life Instrument (WHOQOL- BREF).

The WHOQOL-BREF is an international cross-culturally comparable quality of life assessment instrument [25]. It is available in different languages for both developed and developing countries [26], [27], and it is a generic QOL instrument developed by WHO, and is composed of 26 items. The response options range from 1 (very dissatisfied/very poor) to 5 (very satisfied/very good). It emphasizes the subjective responses rather than the objective life conditions, with assessments made over four weeks. The questionnaire includes four domains: physical health, psychological health, social relations, and environment. The scores are transformed into a linear scale between 0 and 100, with 0 being the least favorable and 100 being the most favorable [25].

### Statistical Analysis

The analysis was divided into three parts. First, we described socio-demographic characteristics of the sample. Secondly, we calculated Cronbach’s α coefficient to determine the internal consistency reliability and factor analysis was carried out using the confirmatory factor analysis (CFA) to test the structural validity of the instrument. CFA was conducted using the software LISREL (version 8.8). We fitted the covariance matrix of the data (24items) with a priori four-factor model in CFA. Finally, QOL of the study sample was compared via t-test and one-way ANOVA according to different years, specialties, gender, and other factors which may have an impact on QOL. Then, Student–Newman–Keuls test was used to make multiple comparisons between the groups.

If the outlier exceeded more than three standard deviations, it was deleted in the statistical analysis. Missing data was included in the analysis and replaced with the median value of all respondents on that question. Since the median value was an integer, it fitted the ordinal scale. The final analysis database was formed after analytical treatment of the data for any logical errors that may exist and for any abnormal values in the data obtained. The data was analyzed using SPSS® version 17.0 (SPSS Inc., Chicago, IL, USA) for Windows®. A P-value of <0.05 was considered to be statistically significant.

### Ethics Statement

All of the participants were well informed about the content and the aim of the questionnaire. The study was an anonymous survey, and the results remain confidential. The questionnaire did not contain any identifying information about the individual subjects. Participation in the study was totally voluntary, and participants had the option of declining to answer specific questions or leaving the entire questionnaire blank if they did not wish to participate. The protocol was approved by the Bioethics Advisory Commission of China Medical University, and all subjects provided written consent before participating in the study. All data remain confidential, and data protection was observed at all stages of the study.

## Results

### Socio-demographic Characteristics of the Population

The average age of students across the entire study group was 22.4±3.1 years. The sample (1686 students) is comprised of 43.1% male and 56.9% female students. No outliers were found in the data collection: the percentages of the missing data were less than 5%, except for item 21“sexual activity” where there was 33% missing data. The social demographic characteristic of the subjects who completed the questionnaires is shown in Table 1.

**Table 1:** Social demographic characteristics of medical students in the study.

Variables	N (%) (n = 1686)	Physical Health Range	Psychological Health Range	Social relations Range	Environment Range
**Gender**					
Male	726(43.1%)	(52.3–81.5)	(49.3–83.6)	(55.6–81.0)	(42.0–80.5)
Female	960(56.9%)	(43.7–87.4)	(41.0–81.6)	(54.3–73.6)	(49.5–78.0)
**Academic year**					
Year 1	382(22.6%)	(45.3–80.1)	(49.3–83.0)	(52.3–86.0)	(55.5–77.5)
Year 2	378(22.4%)	(51.4–85.7)	(50.6–89.6)	(47.6–79.3)	(44.5–76.0)
Year 3	360(21.5%)	(53.1–87.1)	(52.3–89.3)	(51.3–74.0)	(42.0–80.5)
Year 4	363(21.5%)	(40.8–89.6)	(38.6–68.3)	(47.6–80.0)	(38.5–67.0)
Year 5	203(12.0%)	(51.7–79.2)	(54.0–83.6)	(50.6–84.0)	(52.0–79.5)
**Specialty**					
Clinical medicine	1124(66.7%)	(47.1–89.8)	(52.3–92.3)	(54.3–90.0)	(48.5–89.5)
Preventive medicine	272(16.1%)	(49.6–84.3)	(51.0–89.6)	(53.6–90.3)	(54.0–93.5)
Nursing	290(17.2%)	(44.8–86.9)	(52.0–86.7)	(50.6–88.3)	(56.5–90.5)

### Reliability

The degree of internal uniformity among the items was expressed by Cronbach’s α coefficient. The overall Cronbach’s α coefficient of the WHOQOL-BREF questionnaire was 0.731, while the Cronbach’s α coefficient for the physical health, psychological health, social relations, and environment domains were 0.763, 0.794, 0.711 and 0.728, respectively.

### Construct Validity

Structural validity was evaluated by means of factor analysis according to the degree of similarity between the hypothetical structure of the questionnaire conceived by researchers and the actual observed data. Results showed the KMO statistic to be 0.792 and the Bartlett’s spherical check to be χ^2^ = 201.77 and *P* = 0.000, which, when taken together, indicated that the samples in this study were suitable for factor analysis. The results of confirmatory factor analysis indicated that these four domains, physical health, psychological health, social relations and environment, whose characteristic roots were >1, the accumulative contribution rate was up to 69.3% (Table 2). The confirmatory factor analysis (CFA) provided an acceptable fit to the a priori four-factor model when two matching content item pairs were allowed to be correlated; *X^2^* = 697.4, RMSEA = 0.079, NNFI = 0.767, CFI = 0.879. The four first-order CFA showed that the CFI for physical, psychological, social relationships, and environment domains were 0.98, 0.95, 0.96, and 0.93, respectively. All the item loadings of the questionnaire were >0.3, so the results suggested adequate construct validity.

**Table 2:** Item loadings for the four factors of the questionnaire from confirmatory factor analysis.

Item	Physical Health	Psychological Health	Social relations	Environment
3. Pain	**0.67**			
4. Dependence of medical aids	**0.65**			
10. Energy	**0.54**			
15. Mobility	**0.42**			
16. Sleep and rest	**0.37**			
17. Activities of daily living	**0.58**			
18. Work capacity	**0.54**			
5. Positive feeling		**0.57**		
6. Personal belief		**0.68**		
7. Concentration		**0.41**		
11. Bodily image		**0.57**		
19. Self-esteem		**0.39**		
26. Negative feeling		**0.58**		
20. Personal relationships			**0.46**	
21. Sexual activity			**0.52**	
22. Social support			**0.63**	
8. Security				**0.61**
9. Physical environment				**0.43**
12. Financial support				**0.56**
13. Accessibility of information				**0.51**
14. Leisure activity				**0.39**
23. Home environment				**0.41**
24. Health care				**0.44**
25. Transport				**0.42**
% variance	**33.6**	**14.1**	**11.9**	**9.7**

### QOL According to Different Years in Medical Education

**Figure 1:**
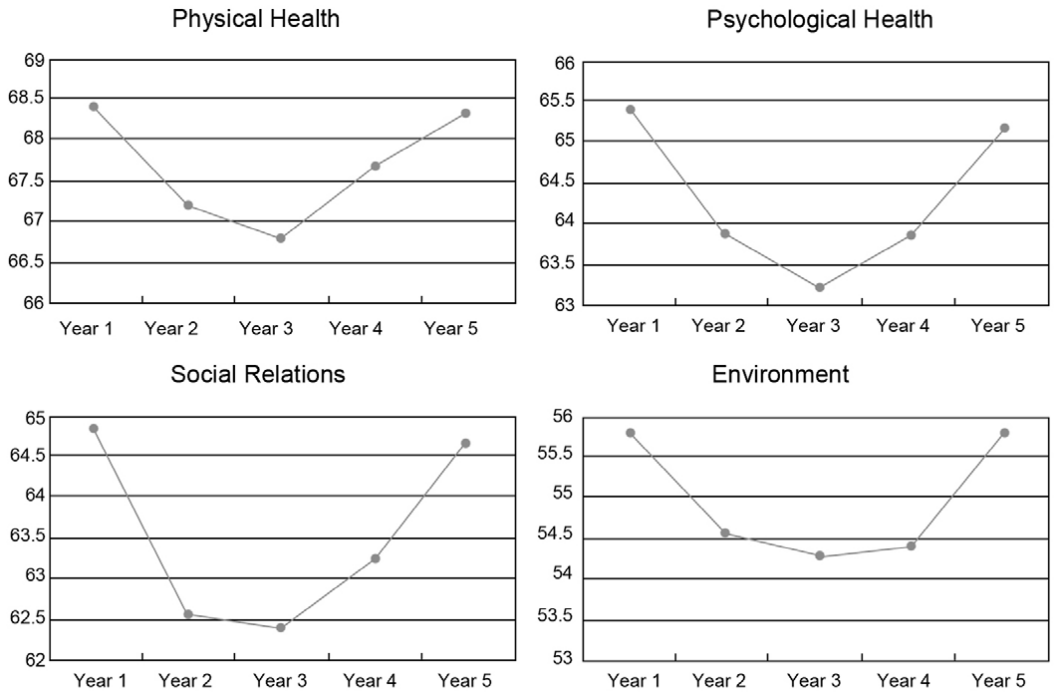
Scores of each domain for medical students.

We found significant differences in the psychological health (F = 5.585, p<0.05) and social relations domains (F = 7.172, p<0.05) according to the different years of study (Table 3). 3^rd^ year students had the lowest scores. The mean scores of each domain in relation to the academic years of study are shown in Figure 1.

**Table 3:** Scores of medical students in different years.

WHOQOL-BREF domains	Grade (* 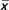 *±*s*)					*F*	*P*-value*
	Year 1	Year 2	Year 3	Year 4	Year 5		
Physical Health	68.45±12.35	67.23±11.89	66.86±13.46	67.59±17.03	68.34±13.22	2.813	0.136
Psychological Health	65.48±14.66^a^	63.91±11.59	63.14±14.12^b^	63.89±12.87	65.12±16.30^a^	5.585	0.031*
Social relations	64.87±14.01^a^	62.54±12.64^b^	62.26±13.74^b^	63.23±10.97	64.68±12.88^a^	7.172	0.018*
Environment	55.78±15.07	54.52±13.97	54.38±12.59	54.45±12.29	55.83±12.03	2.453	0.189

* One-way ANOVA. Mean scores followed by the same letter do not differ according to Student–Newman–Keuls test.

### QOL According to Different Specialties

**Figure 2:**
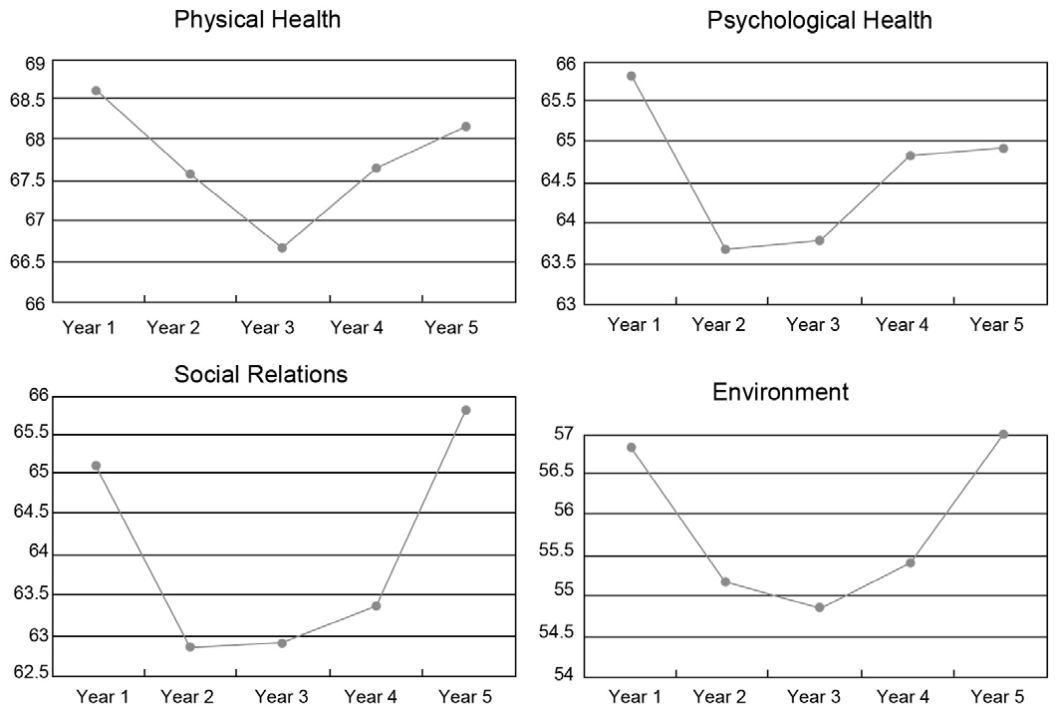
Scores of each domain for clinical medicine.

**Figure 3:**
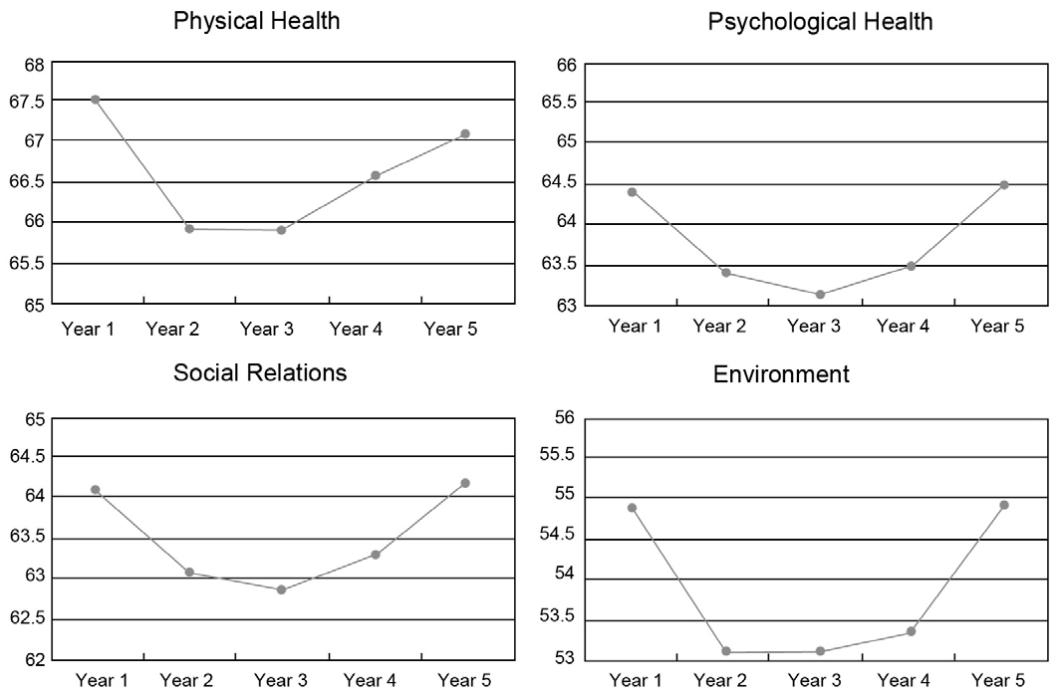
Scores of each domain for preventive medicine.

**Figure 4:**
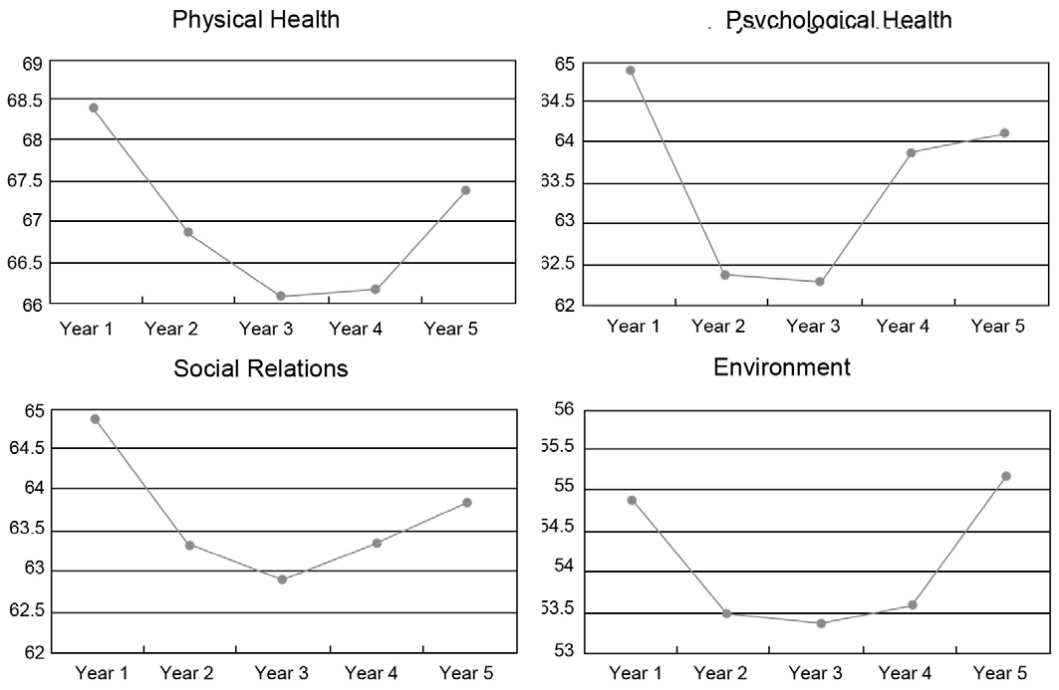
Scores of each domain for nursing.

The scores of different specialties were significantly different in the psychological health and social relations domains. Students from clinical medicine had higher scores than those from other specialties in psychological health (F = 6.788, p<0.05) and social relations domains (F = 4.216, p<0.05) (Table 4). The distribution of the mean scores for each study domain for students in different specializations is shown in Figures 2, 3, and 4, respectively.

**Table 4:** Scores of medical students in different specialties.

WHOQOL-BREF domains	Specialty (* 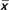 *±*s*)			*F*	*P*-value*
	Clinical medicine	Preventive medicine	Nursing		
Physical Health	68.02±12.88	66.94±12.31	66.88±11.78	2.494	0.185
Psychological Health	64.77±10.35^a^	63.23±13.21^b^	63.03±12.63^b^	6.788	0.017*
Social relations	64.97±13.15^a^	63.37±13.92^b^	63.12±14.71^b^	4.216	0.041*
Environment	55.01±12.69	54.38±12.52	54.98±12.33	1.717	0.452

* One-way ANOVA. Mean scores followed by the same letter do not differ according to Student–Newman–Keuls test.

### QOL According to Impact Factors

Male students had significantly higher scores than female students in the physical health and psychological health domains (p<0.05). Students with different interest levels in the area of study or greater confidence in career development showed significant differences in the physical health and psychological health domains (p<0.05). The scores of students with an urban background were significantly higher than those from rural areas in the psychological health and social relations domains (p<0.05). Students who had more physical exercise had higher scores than students with less physical exercises in four domains (p<0.05) (Table 5).

**Table 5:** Scores of medical students according to the impact factors of QOL.

Variables	N (%)	Physical Health (* 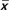 *±*s*)	Psychological Health (* 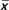 *±*s*)	Social relations (* 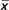 *±*s*)	Environment (* 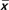 *±*s*)
**Gender**					
Male	726(43.06)	68.59±10.02^a^	65.16±11.34^a^	62.75±12.17^a^	54.89±11.67
Female	960(56.94)	66.93±12.80	63.55±11.92	63.91±10.07	54.96±14.92
**Interest in the area of study**					
Good	595(35.29)	68.31±12.24^b^	64.96±9.89^b^	64.01±11.21	55.39±10.89
General	869(51.54)	67.75±11.97	64.02±10.77	63.13±10.93	54.67±12.78
Insufficient	222(13.17)	65.39±10.24	63.17±12.19	63.07±10.87	54.57±11.19
**Confidence in career development**					
Good	542(32.15)	68.26±10.88^b^	65.61±14.06^b^	64.88±12.17	55.48±10.89
General	876(51.96)	67.55±12.04	63.61±10.97	64.43±12.43	54.71±11.24
Insufficient	268(15.89)	66.63±11.17	63.54±13.15	63.44±11.24	54.51±13.18
**Students hometown location**					
City	897(53.20)	67.82±9.82	65.02±10.78^a^	64.91±10.78^a^	55.11±10.89
rural areas	789(46.80)	67.47±11.05	63.35±12.15	63.84±10.24	54.71±13.18
**The times of physical exercise** **per week**					
0∼1	403(23.90)	66.58±10.67^b^	64.01±10.92^b^	62.48±11.07^b^	53.69±10.93^b^
2∼3	934(55.40)	67.85±11.87	64.08±11.05	63.72±12.76	55.13±12.14
>4	349(20.70)	68.27±11.06	65.78±10.14	63.69±14.21	55.69±11.79

a T-test, p<0.05;

b One-way ANOVA, p<0.05.

## Discussion

The study suggested that the WHOQOL-BREF was reliable and valid to health professionals in the assessment of the QOL of Chinese medical students. The study found that the year of study was confirmed as an important indicator of QOL in medical students (p<0.05). The scores of different specialties are significantly different in the psychological health and social relations domains. Students from clinical medicine had higher scores in the psychological health and social relations domains (p<0.05). Gender, interest in the area of study, confidence in career development and physical exercise influenced the QOL of medical students in different domains.

Reliability was acceptable, with Cronbach’s α coefficient comparable to those reported when using this instrument with a large sample of the general population. The results of confirmatory factor analysis indicated that goodness-of-fit indices were acceptable, and the factor structure of the WHOQOL-BREF was confirmed in this sample. Therefore, the WHOQOL-BREF was valid to use with medical students to assess quality of life in China. The results were consistent with other studies [8], [9]. A similar study in Thailand provided support for using the WHOQOL-BREF for general Thai college students [9]. The percentages of the missing data were less than 5% for all items except for item 21 “sexual activity” (33%). This item involved one’s privacy and people in China are too shy to talk about it with others.

We found that students in 3^rd^ year showed greater impairment in psychological health and social relations as compared with students in other phases of their medical education. As can be seen in Figure 1, the distribution of the scores in the four domains had similar consistency, which followed a “V” shape according to the different academic years. The 3^rd^ year students had the lowest scores in all the domains. Students in different years of study had different workloads. From first year to fourth year, the respective hours of lectures and practical work per year in clinical medicine are as follows: 872 in first year, 892 in second year, 942 in third year, and 798 in fourth year. In fifth year, students undergo a 51-week internship rotational program. Third year students in preventive medicine and nursing also had more academic hours: 888 and 890, respectively. In addition, the curriculum in third year includes many integrated clinical cases, requiring more hours of study. Therefore, there is greater academic pressure on third year students as compared to those in other academic years. The 3^rd^ year represents the transition to the clinical years in China. On the one hand, 3^rd^ year students have more academic courses which involve both basic science and clinical medicine, with the medical curriculum often focusing on disease diagnosis and treatment and paying little attention to education about communication with patients and end of life issues [28]. On the other hand, the 3^rd^ year students are experiencing contact with real patients for the first time. They do not have enough knowledge and skills to interact with patients, especially dying patients [29]. Previous studies reported that students felt overwhelmed, apprehensive, vulnerable and anxious in these circumstances [28], [30]. So the 3^rd^ year becomes the most significant period for the decline of medical students’ QOL scores. A similar study in the Brazil also provided evidence that 3rd year students in the transition to clinical training were under greater impairment [16]. Medical schools in Brazil provide a 6 year program of medical education that follows the traditional curriculum model, and the program is divided into 2 years of basic sciences, 3 years of clinical training and 1 year of internship. Like the 3rd year Chinese medical students, students in years 3 and 4 have their first contact with patients during their transition to clinical years and had lower scores of QOL compared with the students in other years.

For the above issues, medical schools need to take measures to give more support for the 3^rd^ year students. First, medical educators could carry out curriculum reforms that introduce problem-based learning (PBL), integrated courses and early exposure to clinical training. The approach of integrated courses could decrease the repetitive content of the curriculum and give student’s comprehensive understanding of medical courses. Early exposure to clinical training gave students opportunities to relieve the pressure of stress and anxiety in their medical practice and provides more interaction between theoretical and practical disciplines [31]. Research shows that PBL can reduce anxiety and has a positive impact on students’ attitude towards health research for students [32], [33]. These reforms would relieve students’ academic load through balancing the curriculum of basic science and clinical medicine in different years. Secondly, it is very important that we give students more support, which includes enhancing student competency in communication and professionalism and giving students the necessary tools and instruction on how to relieve stress and strain during their medical training [34]. All this could reduce the impairment in physical and mental health of medical students.

Our results show that students in clinical medicine had higher scores in the psychological health and social relations domains as compared to other students. Most medical colleges in China have 5 year programs for different specialties, including clinical medicine, preventive medicine and nursing. Students in different specialties are given similar courses of basic science and clinical medicine from years 1 to 3. The QOL of students in different specialties might be associated with academic load, employment prospects and other individual factors. First, non-clinical students would take more courses than clinical students, including both the essential medical courses and their professional courses. Secondly, some studies show that clinical medical students had better employment prospects with higher salaries and greater respect as compared with non-clinical students in China [3]. Third, the admission standards for clinical students were a little higher than students in other specialties, as reflected in the entrance examination scores. Lastly, we also found that nursing students had the lowest scores in the psychological health domain. Possible reasons were that nurses always feel the anxiety and stress as a result of the poor doctor-patient relationship and great pressure for employment in China.

There are also some impact factors influencing the QOL of medical students in different domains. Male students scored significantly higher than female students in the psychological health domains. Previous studies also provided similar findings [35], [36]. Some researchers have attributed it to females being more emotional and sensitive to pressure [37], [38]. However, female students scored higher than males in the social relations domain. Studies show that women are better than men at dealing with different relationships [39].

In our study, we found that interest in the area of study and confidence in career development had an impact on QOL. Studies show that interest in the area of study was associated with the attitude towards studying [40]. Students with an active study attitude would be more likely to pay attention to their studies and had greater enthusiasm towards their field of study. This would yield better academic performance and benefit the QOL of students in both their physical and mental health aspects. Previous studies show that students with a lower level of interest in their specialty were often plagued by burnout and desperation [41]. In addition, confidence in the career development of these students was directly associated with their employment prospects. Many medical students face great pressures from insufficient job opportunities in China. According to statistics taken by the Chinese government, the increase in number of health personnel every year from 2004 to 2010 was lower than the number of medical graduates [2]. In 2010, nearly half of the 919481 medical graduates, including undergraduates and junior college students of medicine, could not go into their profession of choice directly after graduation.

Medical students from rural areas had lower scores in the psychological health and social relations domains. There are several possible reasons to explain this issue. First, the students from rural areas had to leave their homes and adapt to new life in the city. Simultaneously, they faced a new urban culture shock in this new city life. Evidence was provided in previous studies that students from poor rural areas had lower self-esteem and had some challenges in dealing with social relationships [42]. Compared to their classmates from the city, rural students feel that their experience scope is more limited and that their living situations may be more difficult. In addition, the large cultural gap between city life and rural life made it more difficult for them to deal with social relationships, especially on the account of financial limitations that lead to being unable to attend normal social activities [43]. Medical schools need to pay attention to showing concern for their psychological problems and give them the necessary psychological counseling and financial support. Lastly, the scores of students with the different frequency of physical exercises are significantly different in all four domains. Physical exercise was a positive factor to the QOL of students and it is likely that students would benefit from increased exercise [44].

Although there are some strengths in the study that it provide evidence of the WHOQOL-BREF scores of quality of life in Chinese medical students, the main limitations of this study are: (1) As the sample was drawn from only one school, the population surveyed is not demographically diverse enough to be representative of all Chinese medical students in the mainland and thus generalization of the results must be considered carefully. (2) The study was unable to assess curriculum variable, which must be taken into account for future research. (3) The bio-statistical significances might be due to large sample sizes, so its clinical importance should be considered. (4) The study mainly used univariate analysis, the confounding effect was not considered in the analysis.

### Conclusions

The study results showed that the WHOQOL-BREF performs well for assessing the QOL of Chinese medical students. The transition to clinical training in a traditional curriculum was a period of major impairment to the quality of life of medical students. Students in non-clinical medicine suffered more impairment with academic load and employment pressure. Medical schools need to develop reforms in medical education to relieve the pressure from medical courses and training, and provide students in 3^rd^ year and non-clinical specialties with the necessary support to improve students’ well-being.
